# Proximal Row Carpectomy for Coexisting Kienböck's Disease and Giant Intraosseous Ganglion of the Scaphoid: A Case Report and Review of the Literature

**DOI:** 10.1155/2014/834063

**Published:** 2014-11-03

**Authors:** Miguel Morón, Florian Oellig, Tomás Sánchez

**Affiliations:** ^1^Department of Hand and Plastic Surgery, Kantonsspital Aarau, Tellstraße, 5001 Aarau, Switzerland; ^2^Department of Orthopaedic Surgery, Kantonsspital Olten, Baslerstraße 150, 4600 Olten, Switzerland

## Abstract

The etiologies of Keinböck's disease and intraosseous ganglion remain unknown. Both entities are rare and the coexistence of these two pathologies in the same patient and hand is even less frequent. We report the case of a 40-year-old man with a longstanding history of martial arts practice (karate) who developed an avascular necrosis of the lunate concomitant with a giant intraosseous ganglion of the scaphoid bone successfully managed by proximal row carpectomy. We review the literature of these two diseases.

## 1. Introduction

Idiopathic avascular necrosis of the hand is uncommon and can result in carpal collapse if untreated. Kienböck first described in 1910 [1] ischemia and necrosis of the lunate bone that occur in the absence of an acute fracture or nonunion. Until now the cause of this phenomenon is still undetermined. This disease can also leave sequelae of every wrist arthrosis. Intraosseous ganglion (IOG) cysts are rare benign nonneoplastic bone lesions that have a predilection for long bones of the lower limb (distal tibia or proximal femur) and are not infrequent in carpal bones [2]. The etiology and pathogenesis of the IOG remain unknown and the differential diagnosis is still difficult. There are several publications in the surgical literature regarding the treatment of these two rare entities [3–5]. However, none of these reports describe the surgical treatment for both pathologies simultaneously occurring in the same hand. We report our experience in proximal row carpectomy for Kienböck's disease and giant intraosseous ganglion of the scaphoid bone concomitantly in the same hand of a 40-year-old patient. We review the clinical evaluation, X-ray, CT and MRI appearance of the two diseases, and the surgical treatment.

## 2. Case Report

A 40-year-old right-handed man complained of right wrist pain over 7 years. He performed amateur-level martial arts (karate). There was no history of systemic illness, steroid abuse, inflammatory disease, or specific trauma. He has a history of tobacco abuse (60 pack-years) and worked as a plumber. Physical examination: right wrist: extension 40°, flexion 45°; left wrist: 72° extension, flexion 75°; no pronation restriction was found in either side; Finkelstein test negative.

**Figure 1:**
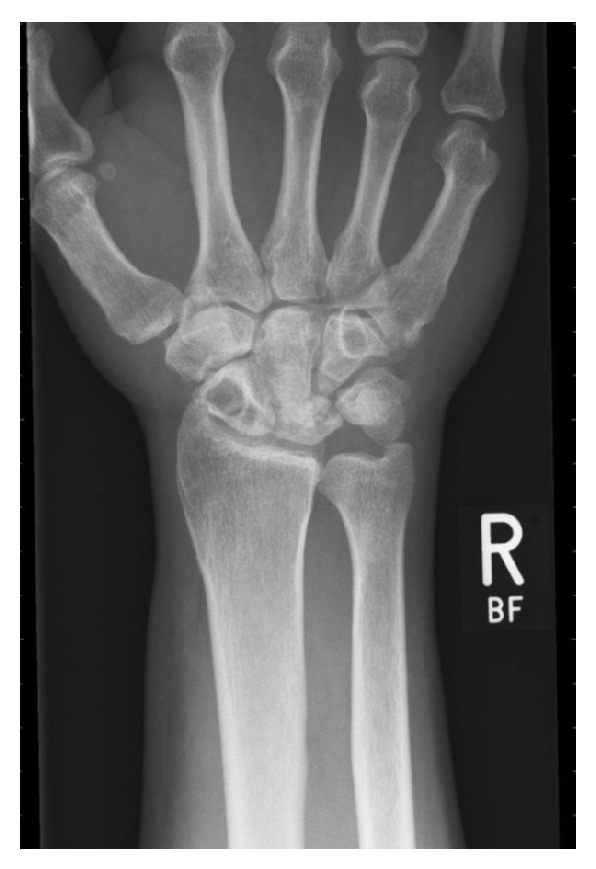
X-Ray: cystic scaphoid changes and partial lunate collapse.

Standard X-ray DR (Figure 1) showed collapse of the lunate with sclerotic changes, cystic appearance of scaphoid marrow with intact cortical borders, and no ulnar variance or no further fracture was present.

**Figure 2:**
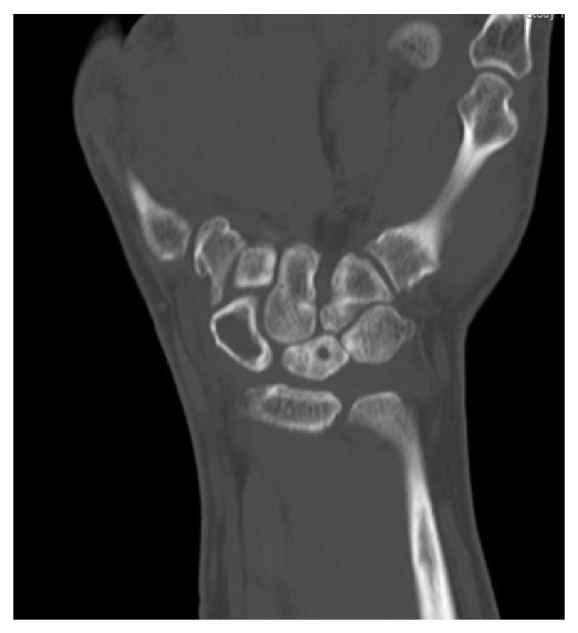
CT: giant interosseous ganglion of the scaphoid and sclerosis of the lunate.

Computed tomography and MRI showed typical signs of Kienböck's disease Stage IIIc with partial lunate collapse and chronic coronal lunate fracture without signs of significant radiocarpal or midcarpal degenerative arthritis (Figure 2). However, a mild subchondral bone marrow edema at the site of fossa lunata and proximal capitate bone was noted.

**Figure 3:**
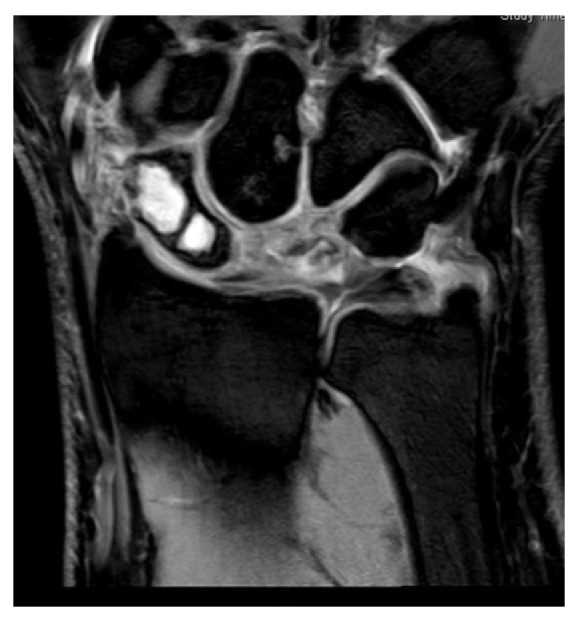
MRI: Giant interosseous ganglion of the scaphoid with cortical bone disrupture, lunate collapse, no severe chondropathy fossa lunata, and capitate.

In addition, there was subtotal displacement of the scaphoid bone marrow by a lobulated cyst like mass that extends palmar to the extraosseous compartment through a cortical defect. These findings are concordant with giant intraosseous ganglion of the scaphoid (Figure 3).

Laboratory tests were normal, ruling out other inflammatory causes.

**Figure 4:**
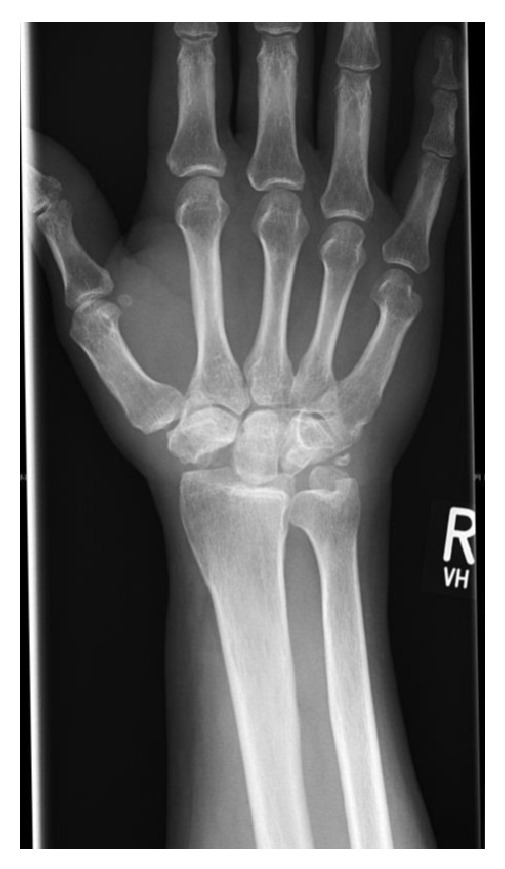
X-ray: postproximal row carpectomy.

On the basis of this diagnosis and due to refractory severe pain that did not respond to conservative treatment, we decided to perform a right proximal row carpectomy (Figure 4).

During the operation, we found fragmentation of both bones (scaphoid and lunate). Capsular closure was performed without difficulty. He had an uncomplicated postoperative course and was subsequently immobilized with casts for 2 weeks.

After 3 months of follow-up, the physical examination in the right wrist showed the following: flexion 30° and extension 30°, and it was pronation- and supination-free. The patient presented no pain and could be reintegrated to his usual work as handcrafter without problems. He could also practice sports without pain and was satisfied with the operation.

**Figure 5:**
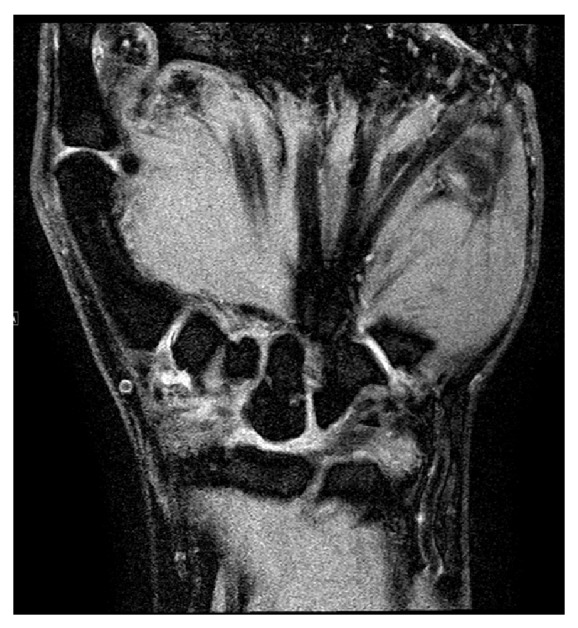
Post-op MRI: no significant chondropathy in fossa lunata or at proximal capitate and no bone marrow edema.

The one-year follow-up MRI (Figure 5) shows a completely resolved bone marrow edema at these sites indicating operative success in terms of nullified overload edema.

## 3. Discussion

Avascular necrosis or osteonecrosis is a common disorder that occurs approximately in 75% of patients between 30 and 60 years and more frequently in men [6]. It can occur in all bones of the body [7] like femoral and humeral heads [8], small bones of the foot, vertebra, ankle, and carpal bones [9]: lunate (Kienböck) [1], scaphoid (Preiser's [10]), pisiform [11], and triquetrum [12].

Aseptic necrosis or idiopathic avascular necrosis of the lunate or most commonly known as Kienböck's disease or lunatomalacia was described first in 1910 [1]. The etiology of the avascular necrosis of the lunate is still unclear, although multiple hypotheses have been suggested including variations in the anatomy and occupation-related repetitive minor trauma. Irisarri [3] analysed in his publication the different aetiologies: traumatic, occupational lunatomalacia, lunate overload, and vascular disturbance, postulating that the cause is more biological than mechanical. The occurrence of a vascular nontraumatic process with a minor infarction pattern in the proximal subchondral area is a plausible hypothesis. He proposed studying the osteogenesis-related genes with polymerase chain reaction (PCR).

The main risk factors are medical conditions including systemic lupus erythematosus [13], smoking, renal disease [14], and scleroderma. More than likely, several predisposing factors exist, that is, the Hultén theory [15] that evaluates the distal radioulnar relationship in normal wrists and in patients with Kienböck's disease. He found that 78% patients with lunatomalacia (17 of 22) presented an ulna-minus stance in a study of Swedish patients compared with 23% in the general population. Also Afshar et al. [16] report an association between ulnar negative variance and the development of Kienböck's disease in the Iranian population. He found that 63% (38 of 60) of Kienböck's disease patients are with ulnar negative stance.

These reports support the hypothesis of an association between the ulnar negative variant and Kienböck's disease. Until now, this theory is controversial because it did not always occur in all patients with Kienböck's disease [3].

Kienböck's disease is more frequently found in young patients between the ages of 20 and 40 years and often without history of acute trauma [17]. These patients are normally manual workers and can suffer repetitive microtrauma leading to degenerative changes of the carpal bones increasing the risk of developing an avascular necrosis of the lunate. Also, these repetitive microtrauma events and Kienböck's disease can occur in athletes as reported by Laframboise et al. [18] about a young varsity football player with negative ulnar variant.

In our case, the hard physical work and sport (karate) are probably affecting the circulation and this in turn can result in a progressive reduction or disruption of the blood supply leading to an avascular necrosis of the lunate. Our hypothesis is supported by the report of Iwasaki et al. [19] on an elderly Kendo (Japanese fencing) player with Kienböck's disease in one wrist and Preiser's disease in the other wrist, suggesting the potential role of sports contributing to these diseases.

Kienböck's patients suffer from insidious, progressive dorsal wrist pain surrounding the lunate with stiffness of the wrist with or without swelling potentially exacerbated by activities.

The reports by Mennen and Sithebe [20], finding that the incidence of asymptomatic Kienböck's disease in the African population was at least 1.9%, are interesting. This study demonstrates that not all cases of avascular necrosis of lunate are symptomatic, dictating the need to investigate more about the natural history of untreated patients.

The diagnosis of lunatomalacia is most frequently established by standard radiographs, although, in the initial stages of the disease, the radiographic changes may be subtle or not present at all.

Progressive changes in the radiographic studies will show linear compression fractures, diffuse sclerosis, cystic changes, lunate collapse, loss of carpal height, and perilunate arthritic changes [16]. The standard X-rays can show also ulna-minus variant and the anatomy of other carpal bones. We preferred not to use bone scans in lunatomalacia because the findings are abnormal but nonspecific. In addition, a computed tomography is recommended to rule out osteoarthritic lesions (Stage IV) and proximally located fractures (Stage IIIA), but the MRI provides more valuable information regarding the distinction between oedema and vascularized and necrotic zone. Schmitt and Kalb [21] report these findings and the important use of intravenous application of gadolinium in MRI to describe the vascular differentiation of the osteonecrotic zone from the reparative zone.

We know that the MRI changes can be severe but, in some cases, they have no correlation with the degree of symptoms.

After doing these radiographic evaluations, it is important to define which stage of Kienböck's disease the patient had before the surgical treatment. We use the Lichtman 4-stage classification system [22], and our case corresponds to Stage IIIc according this classification.

Different techniques are available to replace or ablate the lunate bone, namely, excision and replacement with bone, pronator quadratus, vascularized grafts, or artificial materials such as silicone.

Since the avascular necrosis of the lunate bone is quite infrequent, the treatment options can vary from only observation, wrist denervation, decompression osteotomies, and revascularization procedures to “salvage procedures” like proximal row carpectomy or total wrist arthrodesis.

Lutsky and Beredjiklian [17] suggested different therapeutic approaches, according to the Lichtman stage, as follows: Stage I: cast immobilization for 3 months; Stage II-IIIA with ulnar-negative variance: radial shortening, ulnar lengthening, or capitate shortening; Stage II-IIIA with ulnar-positive variance: vascularized bone graft and external fixation, radial wedge, dome osteotomy, or capitate shortening; Stage IIIB: intercarpal arthrodesis (STT, scaphocapitate), lunate excision, radial shortening, or proximal row carpectomy; Stage IV: proximal row carpectomy (PRC), wrist arthrodesis, or wrist denervation.

Lichtman et al. [22] describe new radiological Stage IIIC with collapse of lunate bone with chronic coronal lunate fracture. There are many surgical options that can be offered for this problem according to Lesley and Lichtman [23] that include lunate excision and STT arthrodesis or PRC.

Pedicled vascularized bone grafts can be used in early stages of Kienböck's disease, when there is no collapse like in Lichtman's Stage II. This technique may also be used with lunate collapse but without scaphoid rotation (Lichtman Stage IIIA), but collapse and persistent nonunion can occur as potential complications [24]. The most common use of the pedicled graft is based on the 4th and 5th extensor compartmental artery (ECA), for which Moran et al. [25] report good results in a retrospective review using these vascularized bone grafts in Stages II, IIIA, and IIIB.

Lesley and Lichtman [23] proposed, in a case of osteonecrosis with lunate collapse (Stage IIIA) with ulnar negative, a distal radial shortening osteotomy and in cases of ulnar neutral or ulnar positive a capitate shortening osteotomy or revascularization procedure. In Stage IIIB with ulnar negative or neutral or positive, they propose scaphoid-capitate or scaphotrapezium-trapezoid arthrodesis or proximal row carpectomy (PRC). Finally, in Stage IV (presence of radiocarpal or midcarpal degenerative arthritis), a salvage procedure such as PRC, total wrist fusion, or total wrist arthroplasty might be necessary.

Intraosseous ganglion cysts are rare benign and lytic bone tumors found in different bones of the body (most frequently distal tibia and proximal femur) [2]. They are also rarely seen in carpal bones, most lesions occurring in the lunate or the scaphoid [5, 26]. Eiken and Jonsson [27] reported 68 out of 80 cases of carpal bone cysts being found in these two bones. The peak incidence varies in the 2nd and 5th decade [5]. Most of them are asymptomatic and are incidental roentgenologic findings. In some cases, they can cause pain.

Physical examination usually reveals tenderness to palpation which may not always be associated with a soft-tissue mass. The aetiology and pathogenesis of this bone lesion remain unknown.

Two theories are proposed: the* penetration theory* postulates that the lesion is originated from adjacent soft tissues with secondary penetration into the underlying bone as reported by Fealy and Lineaweaver [28] in an intraosseous ganglion cyst of the scaphoid and the alternative theory hypothesizes that the process started as an intraosseous degenerative process.

We agree with the Jonsson and Eiken theory [27, 29] of intramedullary vascular disturbance resulting from mechanical stress and repeated minor trauma near the surface of the bone. That means that most intraosseous ganglia arise primarily from within the bone and that communication with the joint is a late event. Roentgenologically, the lesion appears as a small well-defined oval or circular osteolytic lesion with a surrounding area of sclerosis.

Lesions over 5 mm are rare. Most lesions were located in the centre of the carpal bones or close to an articular surface. The eccentrically located cysts were in the scaphoid usually found at the distal end. CT scan is the best X-ray based examination to define the intraosseous lesion [5] but MRI also has the potential to clarify an intra-articular extension or involvement of another carpal bone.

Curettage and bone grafting are the most common surgical treatments in patients with pain and a radiograph revealing an intraosseous ganglion as reported by Peterson [30], Mnif et al. [30], and Javdan et al. [31] in scaphoid localization.

In our patient, we found not only an avascular necrosis of the lunate but also a giant intraosseous ganglion of the scaphoid. In some cases, the differential diagnosis between a giant intraosseous ganglion or an end-stage avascular necrosis of scaphoid (Preiser's disease [32]) can be difficult to ascertain. We supposed that, in our patient, the repeated minor trauma (karate training) provoked an avascular necrosis of the lunate and a giant intraosseous ganglion of the scaphoid. Giant intraosseous ganglia of the scaphoid in association with a collapsing fracture are difficult to treat. In order to make a rational surgical decision in our case, we took into account the following parameters: Kienböck's stage, age of the patient, anatomic and ulna position, and the size and the localisation of the ganglion of scaphoid. One option might be to curette the intraosseous ganglion of scaphoid and bone grafting together with replacement of the lunate bone by silicon prosthesis. But our patient had a giant intraosseous ganglion that would eventually destroy the entire carpal bones and could precipitate a collapse, because it bridges between the proximal and distal carpal rows. Regarding the utilization of a silicon prosthesis, Alexander et al. [33] report in a long term follow-up (average 5 years) study of 10 patients who had lunate silicone replacement arthroplasty for treatment of Kienböck's disease (Stage III) suboptimal results in the short term and further deterioration. The author considered that the use of silicone implants should be undertaken with great caution. The long term outcome results (22–36 years) published by Viljakka et al. [34] confirm that silicone lunate arthroplasty for Kienböck's disease should not be used. Also, Evans et al. [35] reported that silicone replacement arthroplasty in Kienböck's disease is not entirely satisfactory and an alternative more durable material should be used. Instead of using silicone prosthesis, there is currently an alternative pyrocarbon lunate prosthesis for Kienböck's disease IIIB but the experience with this material is insufficient to recommend its use [36].

Lichtman Stage IIIB has carpal instability with radioscaphoid angle greater than 60°, so, initially, we considered a Scaphotrapezial-trapezoidal (STT) arthrodesis as an alternative. But Hohendorff et al. [37] reported in 2012 their prospective study comparing STT arthrodesis with PRC finding slightly better results in patients with PRC compared to STT arthrodesis. Also, Van den Dungen et al. [38] retrospectively compared conservative treatment versus STT arthrodesis for Kienböck's disease with a mean follow-up of 13 years, finding more fractures of the lunate and an increased loss of mobility using the STT arthrodesis.

We acknowledged that using either four-corner fusion or PRC, in our case, would have been good option because they both maintain the grip strength and at the same time achieve pain relief.

Inglis and Jones [39], Imbriglia et al. [40], and Crabbe [41] recommend PRC for cases with late stage of Kienböck's disease since PRC is an acceptable alternative to arthrodesis even when the wrist is likely to be subjected to heavy use.

Our consideration is to remember before using this procedure (PRC) that the articular surfaces of the capitate and radius are intact. In Lichtman Stage IV, the radioscaphoid joint is involved, so a PRC indication must be cautious because of the increased risk of continued pain (Wall and Stern [42]) and risk of early symptomatic radiocapitate degeneration (Croog and Stern [43]). The use of wrist arthroscopy was described by Menth-Chiari et al. in 1999 [44], to identify the articular surfaces within the wrist and to provide the best information of the integrity of the articular surfaces. Bain and Begg [45] have used this procedure for assessment, classification, and recommendation for Kienböck's disease based on the number of nonfunctional articular surfaces.

Traditional radiological staging with CT scan and MRI scan for Kienböck's disease are still not enough to provide more precise radiological information to decide on the best surgical treatment, so the use of arthroscopic grading is recommended frequently.

Interestingly, Budoff [46] described good results after proximal row carpectomy with an interposition flap in a patient with concomitant Kienböck's and Preiser's disease.

## 4. Conclusion

The management of pain and stiffness of the wrist in patients with advanced Kienböck's disease and giant intraosseous ganglion of the scaphoid is a difficult problem. Previous references of concomitant surgical treatments did not exist, but, based on our case, the excision of the proximal row of the carpus is an acceptable and useful alternative procedure.
